# Evaluation of the Mechanical Properties of a 3D-Printed Mortar

**DOI:** 10.3390/ma12244104

**Published:** 2019-12-08

**Authors:** Hojae Lee, Jang-Ho Jay Kim, Jae-Heum Moon, Won-Woo Kim, Eun-A Seo

**Affiliations:** 1 Korea Institute of Civil Engineering and Building Technology, Daehwa-Dong, Goyang-Si, Gyeonggi-Do 10223, Korea; mjh4190@kict.re.kr (J.-H.M.); kimwonwoo@kict.re.kr (W.-W.K.); sea0524@kict.re.kr (E.-A.S.); 2 School of Civil and Environmental Engineering, Yonsei University, Shinchon-dong, Seodaemun-gu, Seoul 03722, Korea; jjhkim@yonsei.ac.kr

**Keywords:** 3D printing, mortar printing, compressive strength, direct tensile bond strength, fabrication variable

## Abstract

The mechanical properties of 3D-printed mortars are determined in terms of their compressive and direct tensile bond strengths. To determine such properties using existing methods, a preliminary experiment was conducted. The compressive strength of the printed mortar was compared to mold-casted specimens and it was found that the compressive strength decreased by ~30%. Among the fabrication variables, an increase in nozzle height negatively influenced the direct tensile bond strength. For the same conditions and age, the direct tensile strength decreased by as much as 16–29% when the number of layers increased from 2 to 6. When the specimens were fabricated using a specially designed stainless steel frame and core drill, followed by extraction and the application of physical impact, the 28 days compressive strength of the specimen decreased by ~50%.

## 1. Introduction

The development of construction technologies using three-dimensional (3D) printing began in the late 1990s, drawing inspiration from the contour crafting technologies developed in the United States [1]. Development of such technologies increased greatly in the 2010’s and has since spread worldwide. Many researchers, including those at Loughborough University [2,3], the University of Surrey [4,5], and Eindhoven Technical University [6], have studied 3D printing for the construction and production of creative structures.

Additive manufacturing (AM) of concrete via 3D printing enables the free-form construction of concrete structures that are irregular in form; much research is ongoing in this area [6,7,8,9,10,11,12]. To secure the structural performance, the methods of evaluating mechanical properties have been extensively explored. For example, Feng et al. [13] evaluated the compressive and flexural strengths of cementitious concrete via binder jet 3D printing. Additionally, Shakor et al. [14] examined the effects of a binder jet device on the mechanical properties of cementitious concrete in terms of its compressive strength.

Concrete 3D printing is advantageous, as it shortens the construction times and enables the convenient construction of irregularly shaped structures. However, the drawback of 3D printing technologies is their low interlayer bond strength. The interlayer bond strength of a structure built via AM has a substantial impact on the performance of the structure, which has served as the focus of many studies, specifically with respect to interlayer bond strength. Le et al. [3] evaluated the effect of printing duration (0 min to 7 days) on bond strength, while Panda et al. [15] investigated the structural bond strength by considering not only the printing gap, but also the nozzle velocity and standoff distance. Furthermore, Zareiyan and Khoshnevis [16,17] evaluated the effects of material changes and the interlocking of interlayers on the mechanical properties of 3D-printed structures.

In this study, we focused on determining the compressive and direct tensile bond strengths among the various mechanical properties of a 3D-printed mortar. The compressive strength of the mortar was evaluated according to the existing standard method (ASTM C 109), and changes in strength were also examined, with the latter depending upon the printing variables, number of layers, printing time gap between layers, and the variation in nozzle height. Finally, we explored the effects of specimen preparation variables on the bond strength of the 3D-printed mortar.

## 2. Materials and Methods

**Figure 1:**
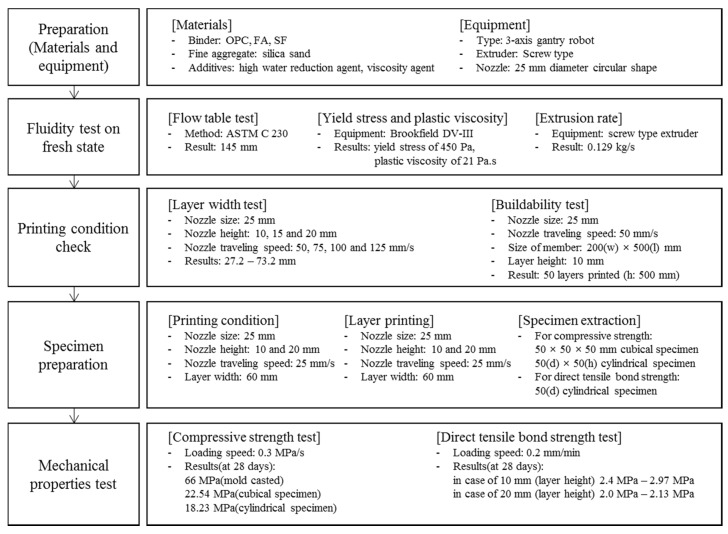
Experimental process for mechanical property evaluation of a 3D-printed mortar.

Figure 1 shows the general research methods employed in this study. After determining the materials and mixing ratio, the properties of the fresh mortar were evaluated. A printing condition test was conducted on the same mortar mixture. Based on the conditions established, the mechanical properties were determined using the prepared specimens and mortar for printing.

### 2.1. Materials

Three types of cementitious materials were used to form the base mixture used in this study: (1) ordinary Portland cement (OPC), (2) fly ash (FA), and (3) silica fume (SF). Type I OPC with a specific gravity of 3.13 g/cm^3^ was used as the major binder. The initial and final setting times of the OPC were determined as 263 min and 360 min, respectively, in the laboratory. Class C FA with a specific gravity of 2.25 g/cm^3^ was used. The loss on ignition of the FA was determined to be 2.5%. Undensified SF with an SiO_2_ content of 91.3% was also used; the percent retained on 45 μm (No. 325) of SF was 4.4%. Sand with a size range of 0.16–0.2 mm was selected when considering the maximum grain size capacity of the pump and the specific gravity of the sand was measured to be 2.59 g/cm^3^. Polycarboxylic acid high-performance water-reducing agent (HWRA) and cellulose viscosity agent were used as the admixture.

### 2.2. Determination of Mixture Proportions for Printing

The extrudability and constructability of the cement-based composite mortar in the experiment were verified using a 3D printing device before printing the specimens. After mixing the materials for printing, the fluidity and rheological properties (i.e., yield stress and plastic viscosity) were evaluated. Along with the proportions of components in the mixture (Table 1), the water–binder (W/B) ratio was 28%. Moreover, the binder replacement ratio was selected based on the pre-test results. Here, factors affecting the tensile bond strength from the usage of various proportions of components were minimized. To prevent material changes that may occur because of the use of various materials, the specimens were manufactured using one type of mixture.

### 2.3. Printing Conditions

#### 2.3.1. Mortar Fluidity and Extrusion Rate Tests

Mortar fluidity, which affects its flow rate, was evaluated via a flow table test [18,19]. In the case of 3D printer extruders, the fluidity of the material affects the extrusion rate. Hence, the extrusion rate and fluidity were simultaneously evaluated via an extrusion test using a screw-type extruder on a 3D printer and a flow table test, respectively. Mortar fluidity was evaluated according to the ASTM International standard method (ASTM C 230) [20]. The fluidity and extrusion rate were measured within 10 min after mixing. The weight of the extruded mortar was measured three times at intervals of 60 s, and then converted into weight per second (kg/s) to calculate the extrusion rate.

#### 2.3.2. Determination of Changes in Layer Widths for Traveling Speed and Nozzle Height

To determine the layer width of the AM mortar, printing was conducted with the extrusion rate and nozzle traveling speed and height as the variables. With the extrusion rate set to 100 rpm, the nozzle traveling speed and height were 50, 75, 100, and 125 mm/s, and 10, 15, and 20 mm, respectively. To determine if the excess load on the upper layer would cause deformation of the lower layer, five layers were printed. The thickness and width were measured for each printed layer and the failure of the upper layer was examined.

### 2.4. Specimen Preparation

#### 2.4.1. Compressive Strength Test

**Figure 2:**
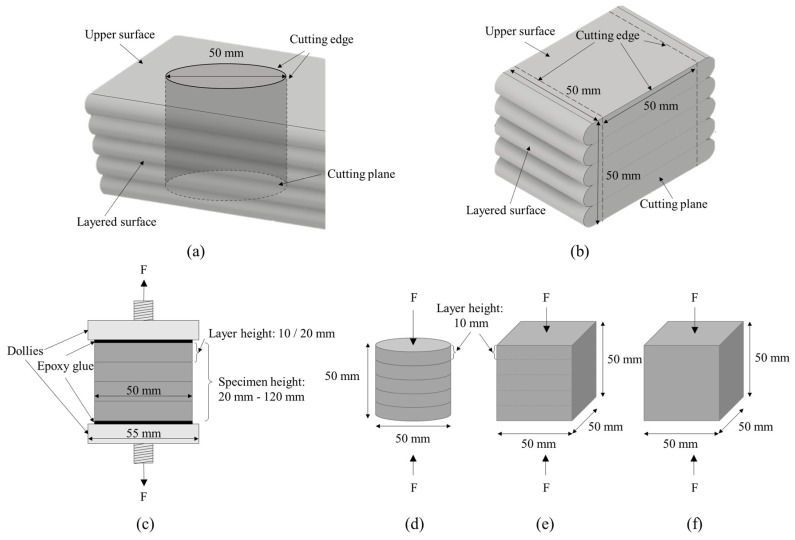
Specimen preparation: (**a**) The fabrication method for a cylindrical specimen from printed layer. (**b**) The fabrication method for cubical specimen from printed layer. (**c**) The printed cylindrical specimen for the direct tensile bond strength test. (**d**) The printed cylindrical specimen for the compressive strength test. (**e**) The printed cubical specimen for the compressive strength test. (**f**) Mold-casted specimen for the compressive strength.

To comparatively evaluate the compressive strength between the AM mortar and a mold-casted specimen, three specimens were fabricated and the compressive strength was evaluated according to the ASTM International standard method (ASTM C 109) [21]. The mortar used for each specimen was mixed using the same mixture proportions. The mold-casted specimen was fabricated using the extruded mortar in the extruder while considering the changes in quality after printing. Mold casting of the specimen was performed using a 50 × 50 × 50-mm cubic mold in accordance with the ASTM C 109 standard [21]. As shown in Figure 2, for the AM mortar, 50 × 50 × 50-mm cubic specimens and Φ50 × 50-mm cylindrical specimens were manufactured 10 min after the printing ended and before hardening began. To evaluate the compressive strength relative to the mold-casted specimen, a cubical specimen and cylindrical specimen were produced in the same manner. 

The mortar printing conditions included five layers printed with the nozzle heights set to 10 mm. To obtain a layer width of 50 mm, printing was performed at a traveling speed of 50 mm/s. To prevent the fluidity and setting time from affecting the strength, printing was completed within 20 min. Ten minutes after printing ceased, the mortar was cut using a 50 × 50 × 50-mm square frame and a Φ50-mm (diameter) cylindrical frame that were specially designed with 0.8-mm-thick stainless steel sheets before hardening began.

#### 2.4.2. Direct Tensile Bond Strength Test

To evaluate the effect of the printing conditions on the direct tensile bond strength, the number of layers and nozzle height were set as variables. With the nozzle height set to 10 mm, 2, 4, 6, 8, and 10 layers were printed to evaluate the correlation between the number of layers and the direct tensile bond strength. The relationship between the nozzle height and direct tensile bond strength was investigated by setting the nozzle height to 20 mm and printing 2, 4, and 6 layers. During printing, the nozzle height was set to 20 mm, the layer width was at least 50 mm, and the travel speed was 25 mm/s using a 25-mm diameter nozzle. To prevent the mortar fluidity and setting time from affecting the bond strength, the same number of layers was printed and the printing process was completed within 20 min. As shown in Figure 2, a 140-mm-high, Φ50-mm (diameter) cylindrical frame was specially designed with a 0.8-mm-thick stainless steel sheet for cutting.

#### 2.4.3. Specimen Fabrication

Tests were conducted to examine the effects of cutting and coring the specimens on their strength before hardening of the mortar. After six layers were printed using a nozzle height of 20 mm and followed by curing for a week, the specimens were extracted using a Φ50-mm (diameter) cylindrical core drill. Specimens extracted 10 min after printing and those that underwent one week of curing and coring were cured in-place.

### 2.5. Compressive and Direct Tensile Bond Strength Tests

#### 2.5.1. Compressive Strength

The compressive strengths of three mold-casted specimens were measured on the 1, 3, 7, 14, and 28 days. The compressive strengths of the four printed specimens were measured each time on the 3, 7, 14, and 28 days. Considering that the compressive strength of the printed specimens could vary depending upon the printing quality, it was measured from four specimens each time to improve the accuracy of the results. Additionally, the compressive strength was measured at a loading speed of 0.3 MPa/s.

#### 2.5.2. Direct Tensile Bond Strength

The direct tensile bond strengths of the five printed specimens were measured each time on the 7, 14, and 28 days. To test the direct tensile bond strengths of specimens, dollies with a diameter of 50 mm and a thickness of 25 mm that could be mounted on a universal testing machine (UTM) were manufactured using stainless steel. The dollies were attached to both ends of each specimen using a high-strength epoxy glue with a bond strength of 26.5 MPa. The direct tensile bond strength was measured at 7, 14, and 28 days, with a displacement control at a loading speed of 0.2 mm/min. To ensure the reliability of test results, five specimens were measured under each condition and on each testing day.

## 3. Results and Discussion

### 3.1. Fluidity and Extrusion Rate

The mixture proportions were determined by applying a viscosity agent at 0.2%. The applied mixture in this study had a rheological yield stress of 420 Pa, a plastic viscosity of 21 Pa·s, and table flow of 145 mm. The extrusion rates were measured three times at intervals of 60 s, and the corresponding mortar weights were 7.94 kg, 7.38 kg, and 7.88 kg (mean: 7.74 kg). The extrusion rate was 0.129 kg/s.

### 3.2. Printing Conditions

**Figure 3:**
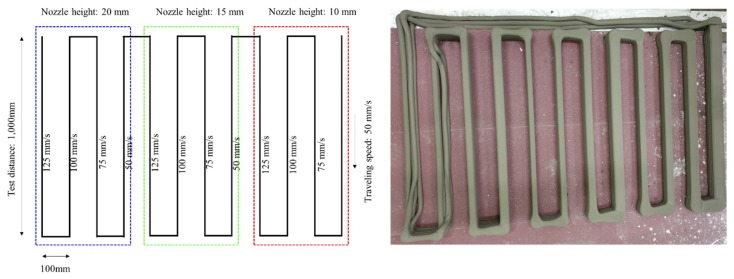
Changes in the layer width under different printing conditions (right), with a schematic (left) detailing the layout of each layer with respect to the printing conditions.

Layer width was evaluated by varying the nozzle traveling speed four times to: 50 mm/s, 75 mm/s, 100 mm/s, and 125 mm/s. Changes in the layer width were also evaluated by changing the nozzle height to 10 mm, 15 mm, and 20 mm. As shown in Figure 3, the layer width changed depending on the nozzle travel speed and height. The width of each layer was measured immediately after printing. The width of each layer for each nozzle height and traveling speed, from the first to the fifth layer in their order of printing from the build platform, are presented in Table 2. Layer widths differed among the layers by up to 1–2 mm, indicating a high level of precision for the printed materials.

**Figure 4:**
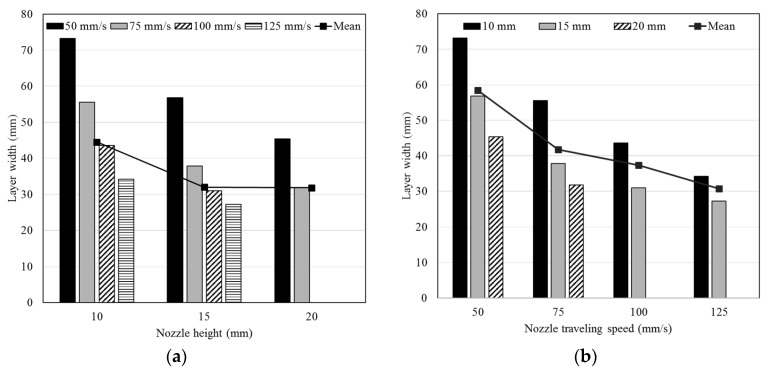
Changes in layer width by varying the (**a**) nozzle height and the (**b**) nozzle traveling speed.

Figure 4 shows the changes in the layer width depending on the nozzle height and travel speed. As the nozzle height increased to 15 and then 20 mm, the layer width decreased by as much as 26% and 40%, respectively, for each 10-mm increase in the nozzle height. As the travel speed increased to 75 mm/s, 100 mm/s, and 125 mm/s, the layer width decreased by as much as 28%, 42%, and 51%, respectively, at intervals of 50 mm/s.

### 3.3. Compressive Strength

**Figure 5:**
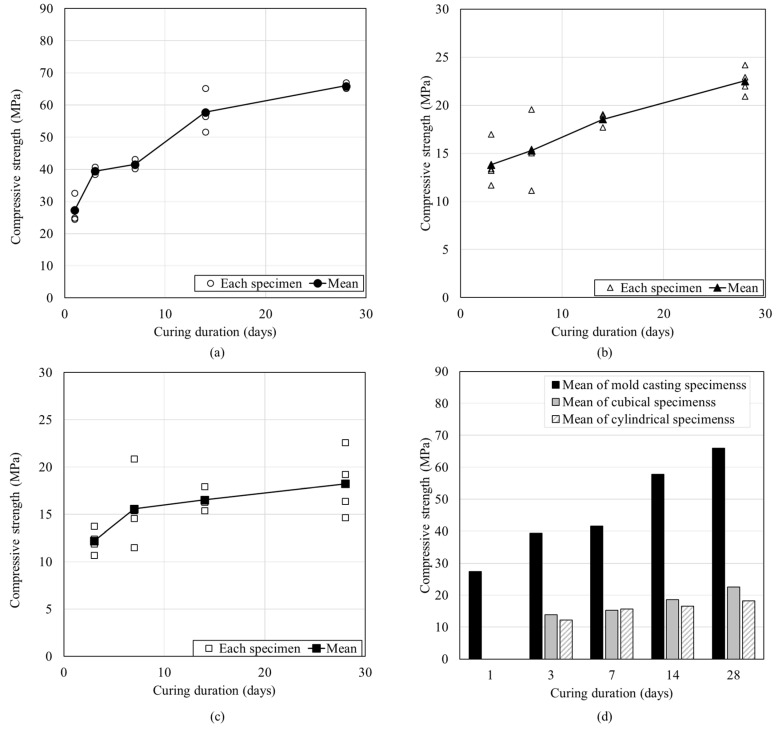
Compressive strength of three different types of specimens: (**a**) mold-casted specimen, (**b**) cubical specimen, and (**c**) cylindrical specimen. (**d**) Comparison of the compressive strengths shown in (**a**–**c**).

To compare the compressive strength of the mold-casted and printed specimens, three types of specimens were produced, and their compressive strengths was measured. Figure 5a shows that on the first day after the mold-casted specimen was produced, the compressive strength was 27.33 MPa, which is ~41% of the compressive strength on the 28th day (66 MPa). Figure 5b shows the compressive strength of the printed cubical specimens, for which the compressive strength on the 3rd day was 13.80 MPa and had increased to 22.54 MPa by the 28th day. Figure 5c shows the compressive strength of the printed cylindrical specimens. The compressive strength on the 3rd day was 12.19 MPa and it increased to 18.23 MPa by the 28th day. Finally, Figure 5d shows the comparison of the compressive strengths of the three different specimens made using the same material. The strength of the printed specimens as ~30% of that for the mold-casted specimens on the 28th day.

### 3.4. Direct Tensile Bond Strength

#### 3.4.1. Number of Layers and Nozzle Height

**Figure 6:**
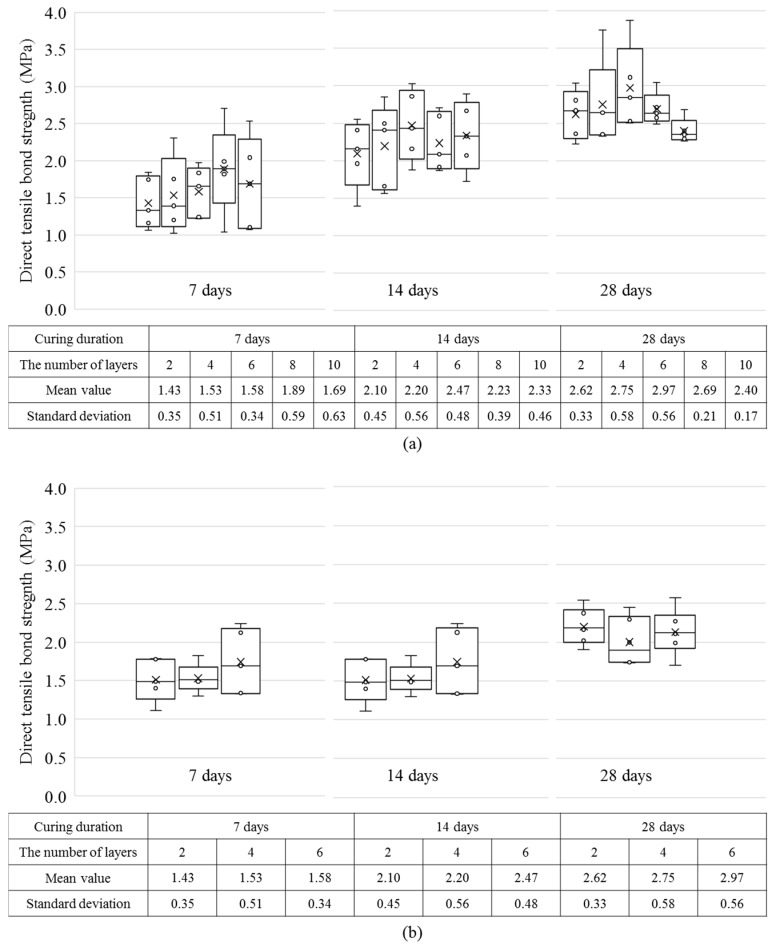
Box-and-whisker plots showing the variation in the direct tensile bond strength because of the nozzle height and number of layers at each age; (**a**) nozzle height: 10 mm, with 2, 4, 6, 8, and 10 layers shown from left to right; (**b**) nozzle height: 20 mm, with 2, 4, and 6 layers shown from left to right.

In Figure 5, the dependence of the direct tensile bond strength on the number of layers when the nozzle height was set to 10 and 20 mm is shown. With an increasing number of layers, the number of interlayers also increased. The effect of this increase on the direct tensile bond strength was examined and the results are shown in Figure 6. Figure 6a shows the direct tensile bond strength of 2, 4, 6, 8, and 10 layers with the nozzle height set to 10 mm. As shown in the figure, the direct tensile bond strength increased in proportion to the age when the number of layers was the same. For example, when the nozzle height was set to 10 mm and the specimen had two layers, the strength continued to increase to 1.43 MPa, 2.10 MPa, and 2.62 MPa with increasing age. However, on the 14th and 28th days, the direct tensile bond strength increased only when the number of layers ranged from 6–8. As the number of layers increased beyond seven, the direct tensile bond strength decreased. 

**Figure 7:**
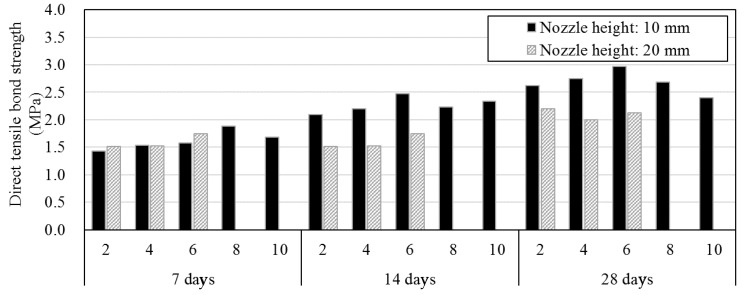
Comparison of the direct tensile bond strength by the nozzle height and number of layers.

**Figure 8:**
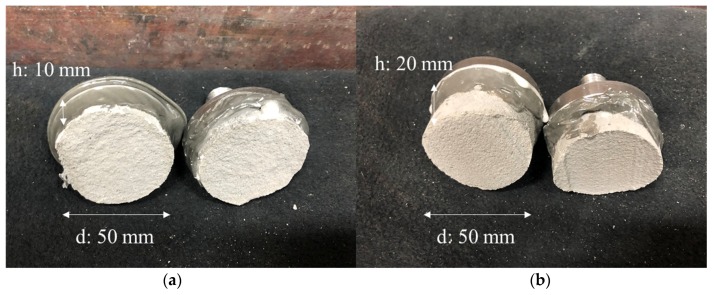
Fractured surfaces after a direct tensile bond strength test with varied nozzle heights: (**a**) 10 mm and (**b**) 20 mm.

Figure 6b displays the direct tensile bond strength of 2, 4, and 6 layers with the nozzle height set to 20 mm. After 7 days of curing, the direct tensile bond strength was similar to that when the nozzle height was 10 mm. In contrast, after 14 and 28 days, the strength was ~70% to that when the nozzle height was 10 mm. Figure 7 shows the mean direct tensile bond strengths for each nozzle height. As is shown, the direct tensile bond strength decreased after six layers with the nozzle height set to 10 mm. However, when the nozzle height was 20 mm, the relationship between the strength and nozzle height could not be obtained since no data were available after six layers. The strength was lower when the nozzle height was 20 mm than when it is 10 mm on the 14 and 28 days of aging. Photographs of the fractured surfaces of the two specimens with two layers are shown in Figure 8.

As shown in Figure 8a, the fracture surface was broken quite irregularly when the nozzle height was 10 mm even though it was the same type of fracture surface. In contrast, the fracture surface was the same as the printed surface when the nozzle height was 20 mm, as shown in Figure 8b. This indicates that when the extrusion rate and nozzle travel speed were the same, the interlayer bonding was weakened as the nozzle height increased.

**Figure 9:**
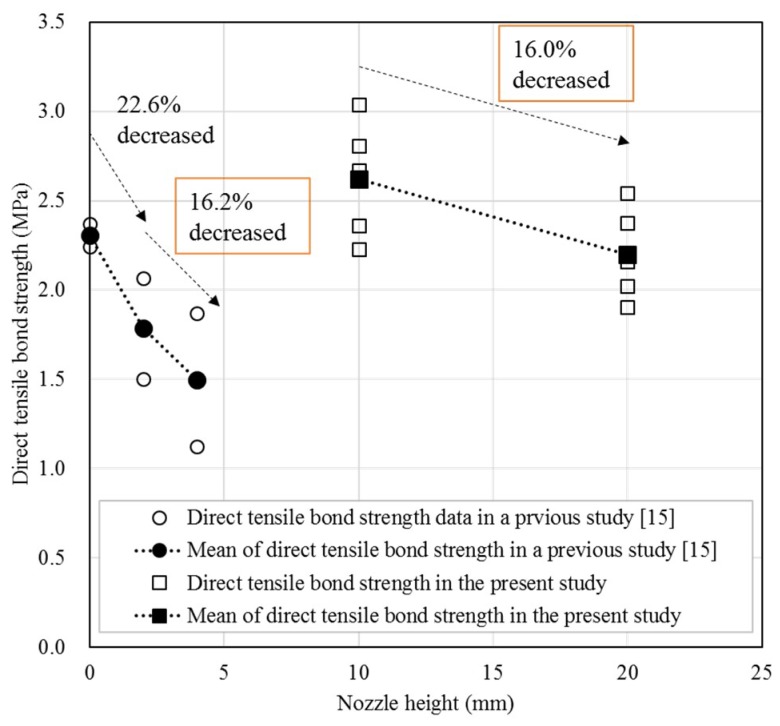
Comparison between previous and current results direct tensile bond strength results.

Figure 9 displays a comparison of the results presented in this study with those of previous studies with respect to the direct tensile bond strength. Specifically, Panda et al. [15] investigated the direct tensile bond strength by printing two layers with nozzle heights of 0 mm, 2 mm, and 4 mm. To compare the results between previous studies to the present study, the strengths of the specimens after 28 days with a two-layer output at 10 and 20 mm were used. As a result, the direct tensile bond strength decreased 16.0–22.6% as the nozzle height was doubled. In particular, the decreasing rates of the direct tensile bond strength with changes in nozzle height between 2–4 mm and 10–20 mm were 16.2% and 16.0%, respectively; it was found that this decreasing tendency in the direct tensile bond strength was similar as the nozzle height increased. However, the materials, equipment, and output conditions applied in the two studies were different, which limits their comparability.

#### 3.4.2. Specimen Fabrication Methods

**Figure 10:**
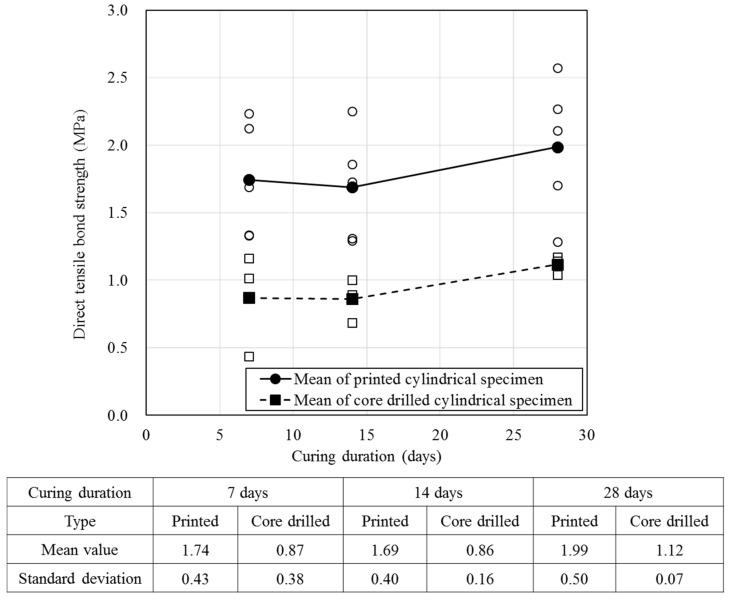
Variation in the direct tensile bond strength with specimen fabrication method.

To measure the direct tensile bond strength, specimens that were cured after cutting using a cylindrical frame (10 min after printing) were tested. The same specimens were produced by core drilling after curing in order to examine the effect of the specimen fabrication method on strength. The strength was measured on the 7th, 14th, and 27th days of aging and the results are shown in Figure 10.

**Figure 11:**
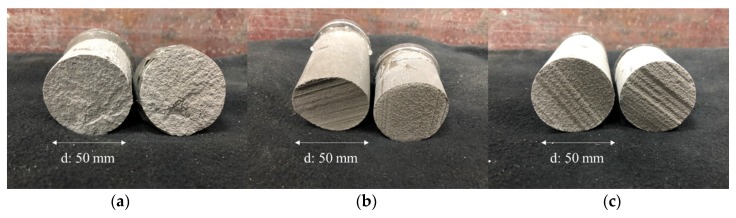
Fractured surfaces after a direct tensile bond strength test used for different nozzle heights and specimen fabricating methods: (**a**) six layers with a nozzle height of 10 mm; (**b**) six layers with a nozzle height of 20 mm; (**c**) six layers core drilled with a nozzle height of 20 mm.

Figure 10 shows the variation in direct tensile bond strength for specimens produced using a stainless steel frame for 10 min after printing with a circular shape. These were produced by core drilling after 1 week of curing with a rectangular shape. The strength of the latter specimens was approximately 0.84–0.87 MPa lower than that of the former specimens, indicating that the strength of the former was only 50–56% of the latter. Figure 11 shows that the fractured surfaces differed depending upon the nozzle height and specimen fabrication method for the same number of layers.

As shown in Figure 11a, the fracture surface was broken quite irregularly when the nozzle height was 10 mm. Meanwhile, in Figure 11b, the area of the surface fracture appears uniform, which indicates that even if the number of layers increased, the bond strength increased as the nozzle height decreased. Finally, the specimen in Figure 11c exhibits a fracture surface of nearly the same shape as shown in Figure 11b. However, the former was fractured with a load of only approximately 50% that of the latter. Although we could not quantitatively measure the impact of the core drill on bond strength, we can expect that the fabrication of the specimen using the core drill had a negative impact on the interlayer bond strength.

## 4. Conclusions

In this study, we evaluated the methods used to prepare 3D-printed mortar, the corresponding mechanical properties, and the factors influencing the properties. When compressive strength was measured using the existing standard methods, the strength of the multi-layered mortar was ~30% that of the mold-casted specimens. Similar results were obtained for cubical and cylindrical specimens; therefore, the reliability of the results is considered to be high. 

The strength of the printed mortar was relatively low because the inside was not as dense as in the mold-casted mortar. In every specimen, strength increased with age, regardless of the number of layers. The relationship between the number of layers and the direct tensile bond strength was nonlinear when the nozzle height was set to 10 mm and strength decreased gradually from the sixth layer. This indicates that, as the number of layers increased, the quality of the interlayer bonding decreased accordingly. As the nozzle height increased, the direct tensile bond strength also decreased. This is because the extrusion pressure on the same contact area of the mortar was lower for the same traveling speed and extrusion rate. It can be inferred that the same tendency will be observed when the nozzle height is 20 mm based on the printing traces of the fractured surfaces.

Specimens produced by core drilling showed that the fabrication method affects the direct tensile bond strength. For 3D-printed mortar, the specimens that can be fabricated are dependent upon the printing device. Meanwhile, coring or cutting may be performed after curing to evaluate the mechanical properties of the printed mortars. These findings indicate that such methods can affect the printed mortar and negatively affect the bond strength, thus limiting its utility in construction projects.

## Figures and Tables

**Table 1:** Proportions of various components in the composite mixture used for printing.

Unit weight (kg/m^3^)						
Water	OPC	FA	SF	Sand	HWRA	Viscosity Agent
232	580	166	83	1146	8.29	1.66

Abbreviations: FA, fly ash; HWRA, high-performance water-reducing agent; OPC, ordinary Portland cement; SF, silica flume.

**Table 2:** Layer widths with a varying nozzle heights and travel speeds.

Nozzle Height (mm)		10 mm				15 mm				20 mm			
Travel Speed (mm/s)		50	75	100	125	50	75	100	125	50	75	100	125
Layer width (mm)	1st	74	56	44	34	58	38	30	26	45	31	25	25
	2nd	73	56	44	36	56	37	30	27	45	31	25	25
	3rd	72	54	43	35	58	39	32	27	45	32	Falling	Falling
	4th	73	56	43	33	56	38	31	29	46	33	-	-
	5th	74	56	44	33	56	37	32	27	46	32	-	-
mean		73.2	55.6	43.6	34.2	56.8	37.8	31	27.2	45.4	31.8	Fail	Fail

## References

[1] Khoshnevis B. (2004). Automated construction by contour crafting—Related robotics and information technologies. Autom. Constr..

[2] Le T.T., Austin S.A., Lim S., Buswell R.A., Gibb A.G.F., Thorpe T. (2012). Mix design and fresh properties for high-performance printing concrete. Mater. Struct..

[3] Le T.T., Austin S.A., Lim S., Buswell R.A., Law R., Gibb A.G.F., Thorpe T. (2012). Hardened properties of high-performance printing concrete. Cement Concr. Res..

[4] Alwi A., Karayiannis S., Starkey B., Gardner M., Reodique K., Varley T. (2013). Construction megascale 3D printing. MegaScale 3D Printing.

[5] Airey J., Nicholls S., Taleb H., Thorley S., Tomlinson S., Hiralal D.U. (2013). Multidisciplinary design project mega scale 3D printing. Mega Scale 3D Printing.

[6] Wolfs R.J.M. (2015). 3D Printing of Concrete Structures. Master’s Thesis.

[7] Perrot A., Rangeard D., Courteille E. (2018). 3D printing of earth-based materials: Processing aspects. Constr. Build. Mater..

[8] Wang X., Jiang M., Zhou Z., Gou J., Hui D. (2017). 3D printing of polymer matrix composites: A review and prospective. Compos. Part B.

[9] Asprone D., Auricchio F., Menna C., Mercuri V. (2018). 3D printing of reinforced concrete elements: Technology and design approach. Constr. Build. Mater..

[10] Balletti C., Ballarin M., Guerra F. (2017). 3D printing: State of the art and future perspectives. J. Cult. Herit..

[11] Ngo T.D., Kashani A., Imbalzano G., Nguyen K.T.Q., Hui D. (2018). Additive manufacturing (3D printing): A review of materials, methods, applications and challenges. Compos. Part B.

[12] Bos F., Wolfs R., Ahmed Z., Salet T. (2016). Additive manufacturing of concrete in construction: Potentials and challenges of 3D concrete printing. Virtual Phys. Prototyp..

[13] Feng P., Meng X., Chen J.-F., Ye L. (2015). Mechanical properties of structures 3D printed with cementitious powders. Constr. Build. Mater..

[14] Shakor P., Sanjayan J., Nazari A., Nejadi S. (2017). Modified 3D printed powder to cement-based material and mechanical properties of cement scaffold used in 3D printing. Constr. Build. Mater..

[15] Panda B., Paul S.C., Mohamed N.A.N., Tay Y.W.D., Tan M.J. (2018). Measurement of tensile bond strength of 3D printed geopolymer mortar. Measurement.

[16] Zareiyan B., Khoshnevis B. (2017). Interlayer adhesion and strength of structures in Contour Crafting—Effects of aggregate size, extrusion rate, and layer thickness. Automat. Constr..

[17] Zareiyan B., Khoshnevis B. (2017). Effects of interlocking on interlayer adhesion and strength of structures in 3D printing of concrete. Automat. Constr..

[18] Kwan A.K.H., Fung W.W.S. (2013). Effects of SP on flowability and cohesiveness of cement-sand mortar. Constr. Build. Mater..

[19] Abebe Y.A., Lohaus L. (2017). Rheological characterization of the structural breakdown process to analyze the stability of flowable mortars under vibration. Constr. Build. Mater..

[20] ASTM (2014). Standard Specification for Flow Table for Use in Tests of Hydraulic Cement.

[21] ASTM (2016). Standard Test Method for Compressive Strength of Hydraulic Cement Mortars (Using 2-in. or [50-mm] Cube Specimens).

